# Calcium oxalate crystals as a cause of multiple punctate echogenic foci in benign thyroid lesions

**DOI:** 10.1007/s10396-024-01448-6

**Published:** 2024-04-25

**Authors:** Ayana Suzuki, Mitsuyoshi Hirokawa, Izumi Otsuka, Akira Miyauchi, Takashi Akamizu

**Affiliations:** 1 Department of Diagnostic Pathology and Cytology, Kuma Hospital, 8-2-35 Shimoyamate-Dori, Chuo-Ku, Kobe, Hyogo 650-0011 Japan; 2 Secretary Section, Kuma Hospital, 8-2-35 Shimoyamate-Dori, Chuo-Ku, Kobe, Hyogo 650-0011 Japan; 3 Department of Surgery, Kuma Hospital, 8-2-35 Shimoyamate-Dori, Chuo-Ku, Kobe, Hyogo 650-0011 Japan; 4 Department of Internal Medicine, Kuma Hospital, 8-2-35 Shimoyamate-Dori, Chuo-Ku, Kobe, Hyogo 650-0011 Japan

**Keywords:** Thyroid, Follicular nodular disease, Graves’ disease, Multiple punctate echogenic foci, Calcium oxalate crystal

## Abstract

**Purpose:**

Multiple punctate echogenic foci (MPEF) on thyroid ultrasonography reflects psammoma bodies in papillary thyroid carcinomas. However, MPEF is also observed in benign thyroid lesions. The aim of this study was to determine the origin of MPEF in patients with benign thyroid lesions.

**Methods:**

We enrolled 26 patients with Graves’ disease (GD) and 24 with follicular nodular disease (FND) who exhibited MPEF and underwent surgery. As controls, we enrolled 40 patients with GD and 32 with FND, but without MPEF, who underwent surgery.

**Results:**

MPEF was observed in both lobes in 80.8% of GDs with MPEF, but was limited to a single lobe in the remaining cases. MPEF was diffusely distributed in 72.3% of the cases and focally distributed in the remaining cases. On ultrasonography, most (92.3%) FNDs with MPEF were solid lesions, and seven nodules (26.9%) were interpreted as intermediate suspicion and their frequencies were higher than in those without MPEF (*p* < 0.01). Microscopically, calcium oxalate (CaOx) crystals were observed more frequently in GDs and FNDs with MPEF (100% and 88.5%, respectively) than in those without MPEF (*p* < 0.001). These differences were particularly significant for CaOx crystals > 100 μm. In GD cases, large CaOx crystals were observed more frequently in the lobes with MPEF than in those without (*p* < 0.05). No psammoma bodies were present in any of the cases.

**Conclusion:**

Appearance of MPEF in GDs and FNDs is not because of psammoma bodies; it is attributable to CaOx crystals larger than 100 μm. Therefore, MPEF is not an indicator of malignancy.

## Introduction

On thyroid ultrasonography, multiple punctate echogenic foci (MPEF) meets the following three criteria: high echogenicity of less than 1 mm, no acoustic shadows, and occurrence of multiple foci [1]. MPEF can be divided into that with and without comet tail artifacts. The former reflects cholesterol crystals in the cystic fluid and is recognized as an indicator of a benign lesion [2]. The latter is known to histologically reflect psammoma bodies present in papillary thyroid carcinoma [3]. The American college of radiology (ACR) thyroid imaging reporting and data system (TI-RADS) and the European TI-RADS describe MPEF as suggestive of malignancy on thyroid ultrasonography [1, 3]. However, we occasionally encounter benign thyroid lesions showing MPEF without comet tail artifacts. To the best of our knowledge, no report has been published regarding this finding to date. Therefore, the aim of the present study was to determine the origin of MPEF in benign thyroid lesions, including Graves’ disease (GD) and follicular nodular disease (FND).

## Materials and methods

### Patients with GD

A total of 1589 patients with GD without any malignant thyroid lesions underwent total thyroidectomy at our institution between 2012 and 2022. Among these, 26 patients (1.6%) showing MPEF on thyroid ultrasonography were included in the study. Ultrasound examinations were performed by ultrasound technologists, and the presence or absence of MPEF was noted on ultrasound reports based on the following criteria: (1) high echogenicity of less than 1 mm, (2) no acoustic shadows, (3) multiple foci, and (4) no comet tail artifacts on ultrasonography. The control group was comprised of 40 GD cases in which total thyroidectomy was performed in 2021, no malignant lesions were microscopically detected in the thyroid, and no MPEF was observed on thyroid ultrasonography.

### Patients with FND

A total of 1209 patients with FND without malignant thyroid lesions underwent thyroid surgery at our institution between 2020 and 2022. Among these, 24 patients (2.0%, 26 nodules) showing MPEF in FND nodules on ultrasonography were included in this study. The control group was comprised of 32 FND cases (41 nodules) in which FND resection was performed in 2021, no malignant lesions were microscopically detected, and no MPEF was observed in the nodules on ultrasonography.

### Data collection

The following clinical and ultrasonographic findings were obtained from the electronic medical records: age, sex, blood and biochemical data, and ultrasound findings. Low and high thyroid-stimulating hormone (TSH) levels were defined as < 0.61 μIU/mL and > 4.23 μIU/mL, respectively. Positivity for levels of antithyroid antibodies including anti-TSH receptor, anti-thyroglobulin, and anti-thyroperoxidase antibodies were defined > 2.00 IU/L, 40.0 IU/mL, and 28.0 IU/mL, respectively. Ultrasonography was performed using an Aplio 80 (SSA-770A; Toshiba Medical Systems Co. Ltd., Otawara, Japan) or an Aplio 500 (TUS-A500; Toshiba Medical Systems Co. Ltd.) ultrasound machine with PLT-805AT (8 MHz, Toshiba) or PLT-1005BT (10 MHz, Toshiba) probes, respectively. The location, composition, echogenicity, and color Doppler findings of FND nodules were examined using ultrasonography. The composition of the FND nodules was classified into three types based on the proportion of the solid area: solid (> 80%), mixed (20–80%), and cystic (< 20%). Samples from representative areas where MPEF was observed were used for histological evaluation of calcium oxalate (CaOx) crystals, cholesterol crystals, psammoma bodies, calcified colloids, calcification, hyalinized stroma, cystic degeneration, granulation tissue, and lymphocytic infiltration. CaOx crystals were observed under a polarized microscope and were classified into two types: small (≤ 100 μm) and large (> 100 μm) crystals. Calcification was classified into three types: micro (< 1 mm), coarse (≥ 1 mm), and capsular calcification [4].

### Statistical analysis

Fisher’s probability test, Mann–Whitney U test, and Pearson’s χ^2^ test were used for statistical analyses, and statistical significance was set at *p* < 0.05.

## Results

### Clinical findings

Table 1 shows the clinical findings in patients with GD and FND, with or without MPEF. The mean age of patients with GD with MPEF was 38.1 years (female/male ratio of 1.36:1) and was not significantly different from that of patients with GD without MPEF. The mean age of patients with FND with MPEF was 58.6 years, which was significantly higher than that of patients with FND without MPEF (49.9 years) (*p* < 0.05). The sex ratio of patients with FND with and without MPEF was not significantly different. Mean TSH levels in patients with GD with and without MPEF were 2.3 μIU/mL and 1.0 μIU/mL, respectively, which was not a significant difference. The mean serum thyroglobulin level in patients with FND with MPEF was 389.6 ng/dL, which was significantly higher than that of patients with FND without MPEF (242.9 ng/dL) (*p* < 0.05). Mean serum calcium levels in patients with GD and FND with MPEF were 9.1 mg/dL and 9.4 mg/dL, respectively, and no patients with hypercalcemia were found in either group.

**Table 1:** Clinical findings in patients with Graves’ disease or follicular nodular disease with or without multiple punctate echogenic foci

	Graves’ disease			Follicular nodular disease		
	With MPEF (26 patients)	Without MPEF (40 patients)	*p*	With MPEF (24 patients)	Without MPEF (32 patients)	*p*
Age: mean [years] (range)	38.1 (12–64)	38.7 (17–69)	0.8235	58.6 (27–76)	49.9 (16–81)	< 0.05
Female/male ratio	1.36:1	3:1	0.1797	3.8:1	15:1	0.1255
TSH						
Mean [μIU/mL] (range)	2.3 (0.003–27.9)	1.0 (0.005–7.32)	0.4826	1.8 (0.005–11.3)	1.2 (0.005–5.4)	0.8296
Low < 0.61 μIU/mL	65.4% (17)	72.5% (29)	0.6966	29.2% (7)	15.6% (5)	1
High > 4.23 μIU/mL	15.4% (4)	10.0% (4)		8.3% (2)	3.1% (1)	
Anti-TSHR antibody	100% (26)	90.0% (36)	0.1475	NA	NA	–
Anti-thyroglobulin antibody	53.8% (14)	40.0% (16)	0.3176	8.3% (2)	21.9% (7)	0.5706
Anti-TPO antibody	92.3% (24)	77.5% (31)	0.1782	9.1% (2/22)	30.0% (6/20)	0.123
Serum thyroglobulin: mean [ng/dL] (range)	2213.9 (32.9–11,592)	1066.4 (1.4–7760)	0.2778	389.6 (9.07–2069)	242.9 (0.96–1921)	< 0.05
Serum calcium: mean [mg/dL] (range)	9.1 (8.2–9.8)	9.2 (8.4–10.0)	0.2563	9.4 (8.9–11.1)	9.5 (8.7–11.4)	0.8296

*MPEF* multiple punctate echogenic foci, *TSH* thyroid-stimulating hormone, *TSHR* thyroid-stimulating hormone receptor, *TPO* thyroid peroxidase

### Ultrasonographic findings

**Fig. 1:**
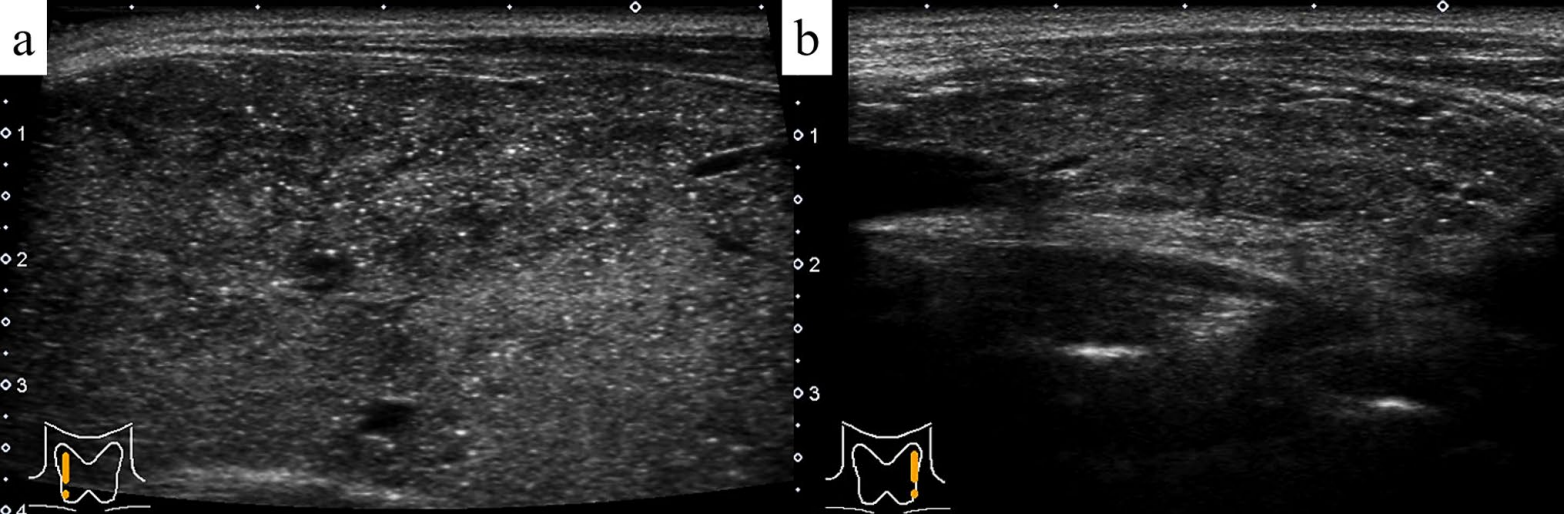
Graves’ disease. Multiple punctate echogenic foci are diffusely present (**a**) and densely concentrated in the lower pole (**b**) (B-mode ultrasonography)

The thyroid volume in patients with GD with MPEF ranged between 37–441 cm^3^ (mean: 182.9 cm^3^) and tended to be larger than that of patients with GD without MPEF (mean: 142.4 cm^3^); however, the difference was not significant. MPEF was observed in both lobes in 21 (80.8%) patients with GD with MPEF, but it was limited to a single lobe (right lobe in two and left lobe in three) in the remaining five patients (19.2%). MPEF was diffusely distributed in 34 lobes (72.3%) (Fig. 1a) and focally distributed in the remaining 13 lobes (27.7%) (Fig. 1b).

**Fig. 2:**
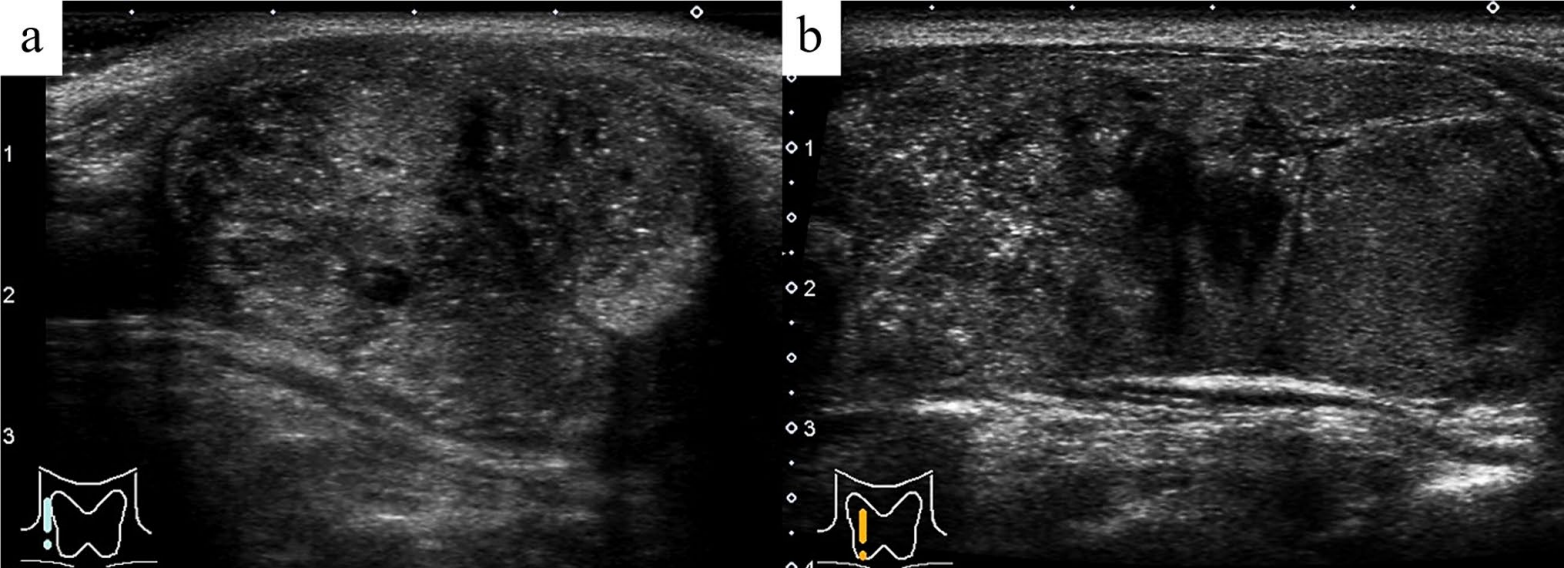
Follicular nodular disease. Multiple punctate echogenic foci are diffusely present (**a**) and localized to the upper pole (**b**) (B-mode ultrasonography)

Table 2 shows the ultrasonographic findings in FND cases with or without MPEF. Eighteen FND nodules with MPEF were localized in the right lobe and eight in the left lobe. MPEF was diffusely distributed in 17 nodules (65.4%) (Fig. 2a) and focally distributed in the remaining nine nodules (34.6%) (Fig. 2b). Twenty-four FND nodules with MPEF (92.3%) were solid, and their frequency was significantly higher than that of the FND nodules without MPEF (52.5%) (*p* < 0.01). FND nodules with MPEF showed significantly higher blood flow than those without MPEF (*p* < 0.05). Seven FND nodules with MPEF (26.9%) were interpreted as having intermediate suspicion, which was more frequent than that in FND nodules without MPEF (2.4%, *p* < 0.01). Five of these were suspected to be papillary thyroid carcinomas, and the remaining two were suspected to be follicular thyroid neoplasms. One FND nodule without MPEF, which was classified as intermediate, was suspected to be a follicular neoplasm. None of the FND cases (with or without MPEF) was highly suspicious. MPEF was not observed in non-nodular areas of the thyroid.

**Table 2:** Ultrasonographic findings in follicular nodular disease with or without multiple punctate echogenic foci

	Follicular nodular disease		
	With MPEF (26 nodules)	Without MPEF (41 nodules)	*p*
Location			
Right lobe	69.2% (18)	36.6% (15)	0.0642
Isthmus	0% (0)	2.4% (1)	
Left lobe	30.8% (8)	61.0 (25)	
Tumor volume: mean [mm^3^]	8.8 (0.04–48.5)	13.0 (0.1–78.0)	0.1786
Distribution of MPEF			
Diffuse	65.4% (17)		
Focal	34.6% (9)		
Composition			
Solid	92.3% (24)	52.5% (21)	< 0.01
Mixed	7.7% (2)	45.0% (18)	
Cystic	0% (0)	2.5% (1)	
Echogenicity			
Hypoechoic	19.2% (5)	15.0% (6)	0.6522
Isoechoic	80.8% (21)	85.0% (34)	
Hyperechoic	0% (0)	0% (0)	
Color Doppler			
−	7.7% (2)	12.5% (5)	< 0.05
±	34.6% (9)	25.0% (10)	
+	42.3% (11)	62.5% (25)	
+ +	15.4% (4)	0% (0)	
Ultrasound report			
Low suspicion	73.1% (19)	92.7% (38)	< 0.01
Intermediate	26.9% (7)	2.4% (1)	
High suspicion	0% (0)	0% (0)	
Undeterminable	0% (0)	4.9% (2)	

*MPEF* multiple punctate echogenic foci

### Histological findings

**Fig. 3:**
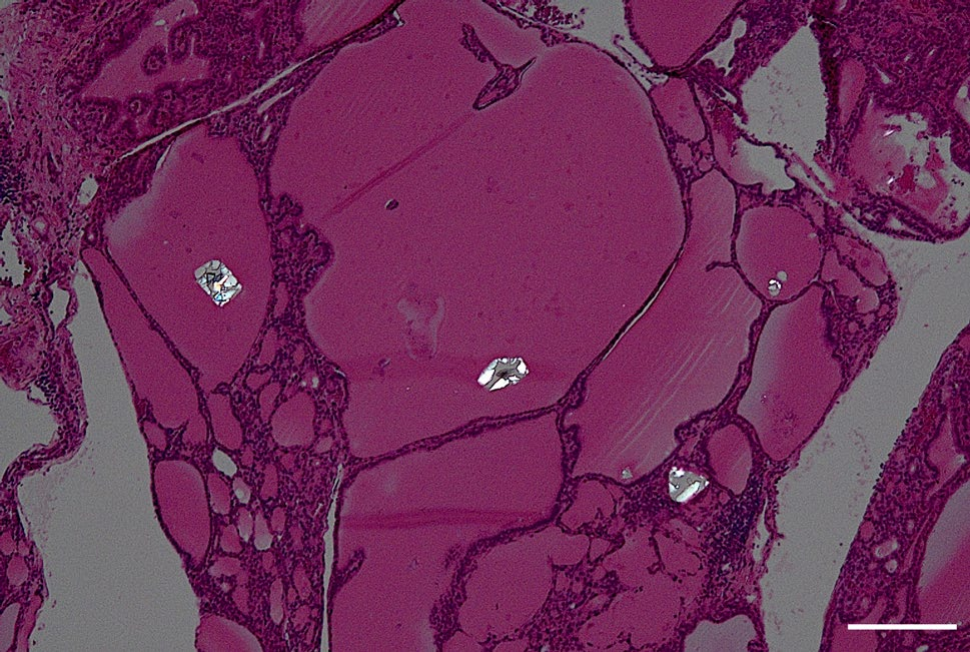
Graves’ disease. A few calcium oxalate crystals are observed in the thyroid follicles. The white bar represents 200 μm (under polarization, hematoxylin & eosin stain, 4 ×)

Table 3 shows the histological findings in GDs and FNDs with or without MPEF. CaOx crystals were observed in all GDs with MPEF, with occurrence rates of 97.9% and 93.6% for small and large crystals, respectively (Fig. 3). CaOx crystals were also observed in 71.3% of GDs without MPEF, with occurrence rates of 71.3% and 11.3% for small and large crystals, respectively. The frequency of CaOx crystals, especially that of large crystals, was significantly higher in GDs with MPEF than in those without MPEF (*p* < 0.00001). In the five patients with GD in whom the distribution of MPEF was limited to a single lobe, the lobe with MPEF exhibited both small and large CaOx crystals, whereas the occurrence rates of small and large CaOx crystals in the lobes without MPEF were 80.0% and 20.0%, respectively. The frequency of large CaOx crystals was significantly different between the lobes with and without MPEF (*p* < 0.05).

**Table 3:** Histological findings in Graves’ disease or follicular nodular disease with or without multiple punctate echogenic foci

	Graves’ disease			Follicular nodular disease		
	With MPEF (47 lobes)	Without MPEF (80 lobes)	*p*	With MPEF (26 nodules)	Without MPEF (41 nodules)	*p*
CaOx crystals	100% (47)	71.3% (57)	< 0.001	88.5% (23)	48.8% (20)	< 0.01
Small	97.9% (46)	71.3% (57)	< 0.001	84.6% (22)	48.8% (20)	< 0.01
Large	93.6% (44)	11.3% (9)	< 0.00001	69.2% (18)	14.6% (6)	< 0.001
Cholesterol crystals	6.4% (3)	2.5% (2)	0.3583	15.4% (4)	26.8% (11)	0.3717
Psammoma bodies	0% (0)	0% (0)	–	0% (0)	0% (0)	–
Calcified colloid	0% (0)	0% (0)		7.7% (2)	0% (0)	–
Calcification	0% (0)	0% (0)	–	11.5% (3)	7.3% (3)	0.6702
Micro	0% (0)	0% (0)	–	0% (0)	2.4% (1)	–
Coarse	0% (0)	0% (0)	–	3.8% (1)	4.9% (2)	–
Capsular	0% (0)	0% (0)	–	0% (0)	0% (0)	–
Hyalinized stroma	0% (0)	0% (0)	–	46.2% (12)	22.0% (9)	0.0579
Cystic changes	0% (0)	0% (0)	–	30.8% (8)	56.1% (23)	< 0.05
Granulation tissue	0% (0)	0% (0)	–	7.7% (2)	12.2% (5)	0.6972
Lymphocytic infiltration	63.8% (30)	56.3% (45)	0.4573	3.8% (1)	12.2% (5)	0.3925

*MPEF* multiple punctate echogenic foci, *CaOx* calcium oxalate

**Fig. 4:**
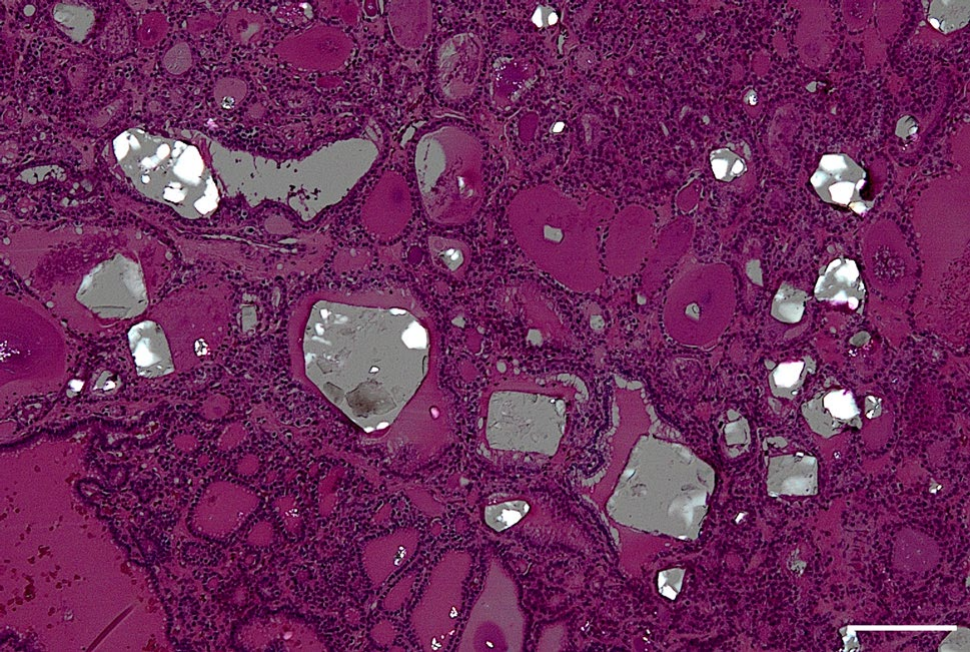
Follicular nodular disease. Mixed small and large calcium oxalate crystals are observed in the thyroid follicles. The white bar represents 200 μm (under polarization, hematoxylin & eosin stain, 4 ×)

CaOx crystals were observed in 88.5% of FND cases with MPEF, with occurrence rates of 84.6% and 69.2% for small and large crystals, respectively (Fig. 4). CaOx crystals were observed in 48.8% of FND cases without MPEF, with occurrence rates of 48.8% and 14.6% for small and large crystals, respectively. The frequency of large crystals was significantly different between FND cases with and without MPEF (*p* < 0.001). Cholesterol crystals were observed in two of the three FND cases, with MPEF and no CaOx crystals.

Cystic changes were also observed in 30.8% of the FNDs with MPEF, but such changes were significantly less frequent than those in the FNDs without MPEF (56.1%) (*p* < 0.05). Cholesterol crystals and lymphocytic infiltration were found in 6.4% and 63.8% of GDs with MPEF, and 15.4% and 3.8% of FNDs with MPEF, respectively, with no significant difference when compared with the respective control. No psammoma bodies were observed in either GDs or FNDs.

## Discussion

MPEF on thyroid ultrasonograms is generally known to reflect psammoma bodies [3, 5]. Psammoma bodies are calcareous bodies with a circular layered structure that are composed mainly of calcium phosphate, and are generally 50–70 μm in size [6, 7]. They are considered a reliable diagnostic feature of papillary thyroid carcinoma [8]. In cases without obvious papillary thyroid carcinoma nodules, the presence of psammoma bodies within the thyroid parenchyma suggests occult papillary thyroid carcinoma [8]. Therefore, MPEF reflecting psammoma bodes is known to be one of the most important ultrasound findings in papillary thyroid carcinoma [1, 3]. This knowledge would likely be the source of the false intermediate suspicion of FNDs in this study. In addition to psammoma bodies, it has also been reported that dystrophic coarse calcification of the capsule and eosinophilic sticky colloid can cause MPEF [9]. Thus, many different possible causes of MPEF need to be considered, although our results suggest that calcifications including psammoma bodies are not involved in MPEF observed in GDs and FNDs.

The only study of MPEF in benign thyroid lesions was reported by Tani et al. [10]. They examined the causative agent of MPEF in various thyroid nodules and observed MPEF in 21.1% and 25.9% of follicular thyroid adenomas and FNDs, respectively. Microcalcifications (≤ 300 μm) in the fibrous stroma, CaOx crystals (50–100 μm), cholesterol crystals (30–200 μm), or calcified colloid in the thyroid follicles (5–100 μm) were suspected to be the causative agents of MPEF. In the present study, we concluded that MPEF was likely attributable to large (> 100 μm) CaOx crystals. This is because large CaOx crystals were observed significantly more frequently in both GDs and FNDs with MPEF than in those without MPEF, and in the lobes with MPEF than in lobes without MPEF in GDs. In addition, no significant differences were found for other substances that cause MPEF.

Our conclusion that large CaOx crystals can cause MPEF raises the following three issues that must be addressed: Firstly, whether the CaOx crystals are hyperechogenic. Secondly, whether ultrasonic device can recognize CaOx crystals as small as 100 μm. Thirdly, the size of the MPEF on ultrasonography was considerably larger than that of the CaOx crystals in the histological specimens. Renal stones composed of CaOx crystals are reported to be hyperechogenic [11]. Taki et al. reported that a 100 μm copper syringe was detectable with a linear probe, although the angle of the probe caused a change in detection capability [12]. Renal stone sizes are consistently overestimated with ultrasonography [13]. These findings would support our conclusion.

CaOx crystal deposits have been reported in various thyroid lesions [14–16]. In GDs, the frequency of CaOx crystal deposition varies greatly from report to report, ranging from 11.1 to 93% [14–16]. In FNDs, the frequency is reported to be 69.4% [16]. In the present study, CaOx crystals were observed in 71.3% and 48.8% of GDs and FNDs without MPEF, respectively. Regardless of their size, CaOx crystals are common in benign thyroid lesions.

However, the mechanism of CaOx crystal deposition in thyroid follicles remains unclear. Richter and McCarty described that the colloidal status might influence the appearance of CaOx crystals [14]. Katoh et al. reported that CaOx crystals were often noted in non-activated follicles that were immunohistochemically T4-negative [16]. Guerlain et al. reported that CaOx crystals tended to occur in the follicles that did not express the transient receptor potential vanilloid 5, a gene involved in Ca^2+^ reabsorption [17]. In this study, CaOx crystal deposits showed heterogeneous distribution. These findings suggest that CaOx crystals may increase under the pathological conditions of follicles. The abnormalities in calcium and oxalic acid transport pathways under pathological conditions need to be studied further.

## Conclusion

The results of the present study suggest that MPEF appearing in GDs and FNDs is not due to psammoma bodies but rather to CaOx crystals larger than 100 μm. Even experienced sonographers seemed to have difficulty distinguishing whether the MPEF was psammoma bodies or CaOx crystals. Therefore, when MPEF is observed on thyroid ultrasonography, the possibility of CaOx crystals and psammoma bodies should be considered. Notably, MPEF alone is not an indicator of malignancy.

## Data Availability

The data supporting the findings of this study are available from the corresponding author upon request.
